# Risk of Single and Multiple Injuries Due to Static Balance and Movement Quality in Physically Active Women

**DOI:** 10.3390/ijerph191912197

**Published:** 2022-09-26

**Authors:** Dawid Koźlenia, Jarosław Domaradzki

**Affiliations:** Unit of Biostructure, Faculty of Physical Education and Sport, Wroclaw University of Health and Sport Sciences, al. I.J. Paderewskiego 35, 51-612 Wroclaw, Poland

**Keywords:** static balance, stability, injury risk, movement, women

## Abstract

Background: Static balance is a reliable indicator of the musculoskeletal and nervous systems, which is a basis for movement stabilization development. The disorders in this area may increase injury risk (IR). This study investigated the musculoskeletal injury risk due to static balance and movement quality regarding single and multiple injury occurrences in physically active women. Methods: The study sample was 88 women aged 21.48 ± 1.56. The injury data were obtained with a questionnaire, and Deep Squat (DS), In-line lunge (IL), and Hurdle Step (HS) tests were conducted. Static balance was assessed with a stabilometric platform measured center of gravity area circle (AC) and path length (PL) with open (OE) and closed eyes (CE), maintaining a standing position for the 30 s. Results: The logistic regression models revealed the general injury occurrence was predicted by AC-CE (OR = 0.70; *p* = 0.03) and IL (OR = 0.49; *p* = 0.03), and the two-factor model AC-CE*IL, (OR = 1.40; *p* < 0.01). When the single injury was predicted by the same factors AC-CE (OR = 0.49; *p* < 0.01), IL (OR = 0.36; *p* = 0.01), and AC-CE*IL (OR = 1.58; *p* < 0.01). Conclusion: Static balance and movement stability predict musculoskeletal injury risk alone and in one model. A further study is needed to verify the efficiency of indicated factors in prospective terms. Using both quantitative and qualitative tests could be helpful in IR prediction.

## 1. Introduction

Static balance is one of the most credible indicators of the musculoskeletal and nervous system state, which states the ability to maintain an upright posture and to keep the line of gravity within the limits of the base of support [1,2]. It expresses the harmonious work of the nervous and musculoskeletal systems. The disorders associated with balance are mainly observed in the elderly and are related to involution, but young ones can also have a problem [3]. Static balance disorders worsen the stabilization during the movements, which causes additional energy expenditure, compensation, and mobility limit. Therefore, movement becomes less effective [4,5,6]. However, this association is poorly explained [7].

High-quality movement patterns require a high level of mobility, stability, and motor control [4,5,6]. It was shown that there is a relationship between functional movement and postural stabilization [8]. The correct movement pattern is performed with the lowest energy expenditure, ensuring the effectiveness and precision of motor activity with safety for tissues [9,10]. Despite generally the same principles of performance, individual movement acts differ in humans. The movement pattern is understood here as a unique way of realizing a specific movement act [11].

Both balance and movement patterns quality are considered injury risk factors [12,13,14,15]. It was shown that athletes with lower stability are more likely to be injured. Especially cruciate ligaments are at injury risk [16]. It could be more evident in women who are more likely to have knee valgus; therefore, it is more probable for injury occurrence [17]. Moreover, movement pattern quality state a valuable factor in injury risk. Chorba et al. [18] indicated that female athletes with poor movement patterns are more likely to be injured.

However, less is known about the usefulness of the single Functional Movement Screen (FMS) module test in injury risk prediction. Especially in terms of combining its results with other measurements. Hartigan et al. [19] investigated the in-line lunge (IL) scores test with balance ability and sprint and jump performance. However, there is a lack of data considering IL results with injury. Deep squat (DS) screening could be considered an injury risk predictor [20]. Hurdle Step (HS) is regarded as associated with physical performance [21], but results provided by Shimoura et al. [22] indicated DS and HS as injury risk factors.

The above observations lead to questions about the possibility of using balance measurements and movement pattern tests based on stability in injury prediction. However, the accessible data mainly focus on one-factor analysis, omitting interference with other attributes that do not state the whole picture of human body property expressed to movement ability. There is a need to make an analysis concern more factors and examine the interaction between them. Therefore, this study aimed to investigate the injury risk due to static balance and movement quality and the single and multiple injury occurrence in physically active women. Specifically, we wanted to answer the following: (1) Which parameters differ in injured and uninjured subjects? (2) Which measured static balance parameters and movement patterns scores predict injury risk: (3) Is there the possibility of creating a two-factor prediction model in injury risk based on static balance and movement patterns quality? Moreover, (4) we deepened the analysis for injury regarding single and multiple incidents. The obtained results allow for a deeper look at the structure of dependencies between human movement abilities, enabling effective injury prediction and targeting adequate actions for injury prevention in physically active women.

## 2. Materials and Methods

### 2.1. Ethics

The relevant study followed the ethical Helsinki Declaration and was approved by The Senate Research Ethics Committee at the Wroclaw University of Health and Sport Sciences (ECUPE 16/2018).

### 2.2. Participants

Participants were volunteers recruited from female students in the faculty of physical education and sport. The inclusion criteria were: (1) no experience in professional sport; (2) no injury 28 days before the measurement or any other medical contradiction for physical activity; (3) high level of physical activity based on IPAQ principles [23]—more than 1500 MET gained from 3 days of vigorous effort; or more than 3000 MET gained from 7 days of vigorous and/or moderate effort. Initially study involved 105 participants, but 17 did not complete the study due to the following: injury 28 days before the survey or other medical contradiction for physical activity (*n* = 3), not conducting all measurements (*n* = 4), and rejection from participation without no giving any reasons (*n* = 7); therefore, data of 88 subjects were included in the final analysis. The examined women were 21.48 ± 1.56 years, body height 1.68 ± 0.06 cm; body weight 60.5 ± 9.00 kg; BMI 21.48 ± 2.52 kg/m^2^; their sports experience was between 4 and 12 years, and weekly training volume was 3–12 h per week. The physical activity level based on IPAQ results was 4535.36 ± 2897.91 MET. According to the IPAQ criteria [23], all participants were physically active; 40.90% of women were injured, 13.63% once, and 27.27% had more than one injury.

All participants were volunteers and were asked to sign a written consent before participating in this study. All subjects were informed in detail about the aim, methodology, and participation conditions. They could withdraw from the research at any moment without providing any reason.

### 2.3. Settings

The research was carried out in the Biokinetics Research Laboratory of the Academy of Physical Education, which has a Quality Management System Certificate PN-EN ISO 9001: 2009 (Certificate Reg. No. PW-48606-10E).

### 2.4. Measurements

The body height and mass were measured with anthropometer SECA model 764 (Seca GmbH & Co. KG, Hamburg, Germany). Based on obtained values, the body mass index was calculated.

Injury data of musculoskeletal system injuries that occurred during physical activity were gained with the Injury History questionnaire (IHQ). This tool was validated with an alpha-Cronbach coefficient at level 0.836, indicating high reliability [15]. The IHQ includes questions concerning the number of injuries concerning the body part (head, neck, torso, upper and lower limbs). This study analyzed the total amount of injuries. The survey was conducted in a supervised manner. The researcher was available to the respondents during the survey.

The International Physical Activity Questionnaire (IPAQ) described the physical activity level. This questionnaire provides [23] measures and assesses the self-reported information about the average time spent on physical activity (minutes per week) and is used among young, middle-aged adults, and nonprofessional sports people. The obtained data allow calculating the number of Metabolic Equivalents of Task—MET. With this questionnaire, we also asked about trained sports, the average number of training, and the duration of a single session.

Static balance was measured with ACCU SWAY stabilometric platform—with Balance Cline software (Advanced Mechanical Technology, Inc. [AMTI], Newton, MA, USA). The participants stood on the platform without shoes, with the upper limbs lowered along the torso. Firstly, they were asked not to move and maintain a standing position for the 30 s with open eyes, and next, under the same condition but with closed eyes. The parameters included in the analysis were the distance along the center of gravity and the field’s perimeter determined by the center of gravity path traveled during the measurement.

The Functional Movement Screen (FMS) is used in movement pattern screening. In this study, three movement tests requiring leg stance were chosen, which involved proper stability and balance: deep squat (DS), hurdle step (HS), and in-line lunge (IL). All movements were assessed on a scale of 0–3, where 0 means pain during the movement, 1 is a lack of ability to move, 2 is moving with compensation, and 3 is moving without compensation. HS and IL are unilateral tests; therefore, they are performed for both body sides, and the worse score is considered [6].

### 2.5. Statistics

The means, standard deviations, and confidence intervals were calculated for normal distribution data and the median with standard errors for data lacking normal distribution. The Student’s *t*-test comparison was made for static balance results and the U Mann–Whitney test for movement quality tests. When the injury occurrence was respected (no injury; single injury; multiple injury group), the Kruskal–Wallis test and Dunn test (post hoc) were conducted to determine the static balance and movement quality differences in those groups. The logistic regression models were built to assess injury risk due to general, single, and multiple injury occurrences based on factors differentiative between groups. Then, in the second step of the analysis, the two-factor logistic regression was conducted. In both, Wald’s Statistics were provided. The reference group stated subjects with no injury. The level of significance was set at *p* < 0.05. Statistica 13.0 (Statsoft Poland, Cracow, Poland) was used for analysis.

## 3. Results

Table 1 presents the *t*-test comparison of static balance test results between the no injury and injury group. It was revealed that the injured group had statistically significantly worse area circle closed eyes scores than the no injury group. Moreover, the path length closed eyes scores difference was close to statistically significant.

Table 2 shows the results of the U Mann–Whitney test comparison of movement quality test results between the no injury and injury group. It was revealed that the injured group had statistically significantly worse in-line lunge scores than the no-injury group. No other tests were statistically different.

Further, the analysis of differences was respected to no injury group, single injury (one), and multiple injuries (more than one), so the Kruskal–Wallis test was conducted with the post hoc Bunn test (Table 3). It was revealed that the area circle closed eye (H = 12.97; *p* = 0.015) differed between the group with no injury with both injured groups. Area circle path length (H = 6.48; *p* = 0.0391) varies only between no injury and multiple injuries (Table 3). Regarding movement patterns quality, only in-line lunge differs (H = 10.08; *p* = 0.0065) between no injury and both injured groups (Table 3).

Table 4 presents the logistic regression models for injury risk regarding injury occurrence (general, single, multiple). In the case of a general and single injury, both factors AC-CE and IL predict injury risk. The two-factor model showed that the injury risk increases with a decreased static balance and worsening movement quality. However, when multiple injury occurrences were predicted, no model was credible due to the lack of statistical significance.

## 4. Discussion

The results indicate the possibility of predicting injury risk based on static balance and movement quality in physically active women. In these terms, measurement of center of pressure area circle closed eyes (AC-CE) and in-line lunge (IL) screening seems helpful in injury risk detection. It also indicated the possibility of deepening the analysis of Functional Movement Screen results to the overall score and single module tests. Moreover, using both tests together seems more effective in injury risk prediction, but not for multiple incidents that were not predicted reliably.

Static balance is considered an indicator of good work of musculoskeletal and nervous systems [2]. It is also considered an injury risk factor due to lower balance ability in injured subjects [24,25]. Oshima et al. [14] provided the observations in a prospective study that indicated static balance as a significant factor in the anterior cruciate ligament in collegiate athletes. It showed that the one injury risk factor is postural control function. In the following study, these results were also confirmed [16]. Moreover, Hrysomalis [12] showed that injury might occur due to poor balance, suggesting improving deficits in this ability as effective injury prevention. Dunsky et al. [1] also indicated that improving static and dynamic balance is an effective injury prevention method.

Therefore, the above observation suggests that other factors could have a role in injury mechanisms. The other one is movement quality screening [13,15]. The most reliable is the FMS test, which is the tool that is helpful in injury risk prediction in the physically active group [26]. Injury risk assessment is mainly based on the overall score, but some results also suggest the usefulness of a single module test [20,22]. In our study, the IL test predicts injury in the general approach and due to a single injury. However, Shimoura et al. [22], in the study on male basketball players, indicated that deep squat (DS) and hurdle step (HS) tests were injury predictors. The difference in our observation may be due to sex and sports group. Moreover, Bunn et al. [20] also pointed out that deep squats as a single screening test could be helpful in injury prediction. Deep squats, hurdle steps, and in-line lunges are global movement patterns based on motor control, mobility, and stability [6]. Therefore, using them together with static balance examination may provide a deeper insight into the balance disorders and associated injury risk.

Our results showed that two-factor models based on COP AC-CE and IL predict injury risk in a general approach and single occurrence. Some results suggested that balance ability is strongly associated with IL score, which may explain that both factors predict injury risk [27]. The results of a study by de la Motte et al. [28] suggested using movement patterns screening with balance examination together as a more reliable way to predict injury than alone. However, the results published by Lisman et al. [29] cannot be omitted, which provided opposite results that indicated the need for further investigation. However, there is a need to emphasize the different methodology because we used the static balance platform. In contrast, the results mentioned above consider results from the y-balance test, which also measures stability with another view. Studies examining injury risk factors state a vital contribution in sports science, worth developing and exploring. However, especially valuable are researches considered more than one factor [30].

We are aware of this study’s limitations. Balance is a complex ability associated with vestibular, visual, and proprioceptive systems, and evaluation of its efficiency would provide more complex and reliable results. There was no examination of body posture (e.g., foot arch, pelvis position, spine shape, etc.), which may provide more related data. Moreover, static balance underrepresents postural control demands in daily life, so there is a need to interpret these results as a part of balance ability. We investigated only women of narrow age, indicating a strong need for further study to examine the similar association in men and other age groups. The group was not heterogenous according to sport discipline. More, we did not verify provided results in perspective terms. Therefore, there is also a strong need to conduct this kind of study to describe the efficiency of indicated parameters as an injury risk predictor.

## 5. Conclusions

The static balance ability and movement quality (alone and jointly) predict injury risk in physically active women. The most reliable parameters seem to be the center of pressure area circle closed eye, and in-line lunge test, which indicate injury alone and in one model. However, those parameters did not predict multiple injury occurrences. Using quantitative and qualitative measurements together to screen injury risk could be helpful in injury risk detection. However, the factors mentioned above should be used with caution. There is a need to verify them in prospective terms.

## Figures and Tables

**Table 1:** Student’s *t*-test comparison static balance test results.

Variable		General	No Injury	Injury	t	*p*
		Mean ± SD	Mean ± SD	Mean ± SD		
		(95% CI)	(95% CI)	(95% CI)		
Static balance	Area Circle	2.14 ± 1.38	1.96 ± 1.16	2.41 ± 1.63	−1.51	0.1335
	Open Eyes	(1.85–2.44)	(1.64–2.28)	(1.86–2.96)		
	Path Length	3.72 ± 1.73	38.89 ± 6.05	40.05 ± 8.96	−0.72	0.4678
	Open Eyes	(3.36–4.09)	(37.20–40.57)	(37.02–43.08)		
	Area Circle	50.43 ± 9.25	3.26 ± 1.77	4.39 ± 1.45	−3.18	0.0020 *
	Closed Eyes	(48.47–52.39)	(2.77–3.75)	(3.90–4.89)		
	Path Length	39.36 ± 7.35	48.88 ± 10.19	52.66 ± 7.26	−1.91	0.0588
	Closed Eyes	(37.8–40.92)	(46.05–51.72)	(50.21–55.12)		

* Statistically significant *p* < 0.05.

**Table 2:** U Mann–Whitney test comparison of movement patterns quality tests results.

Variable		General	No Injury		Injury		z	*p*
		ME ± SE	ME ± SE	Ranks	ME ± SE	Ranks		
Movement Patterns Quality	Deep Squat	2 ± 0.08	2 ± 0.11	2521	2 ± 0.11	1395	1.75	0.0796
	Hurdle Step	2 ± 0.06	2 ± 0.07	2321	2 ± 0.10	1595	0.05	0.9560
	In-line lunge	2 ± 0.07	2 ± 0.08	2651.5	2 ± 0.13	1264.5	2.86	0.0042 *

* Statistically significant *p* < 0.05.

**Table 3:** Post-hoc (Dunn test) results.

Variable	No Injury vs. Single Injury		No Injury vs. Multiple Injuries		Single Injury vs. Multiple Injuries	
	z	*p*	z	*p*	z	*p*
Area Circle Closed Eyes	2.64	0.0245 *	3.01	0.0077	0.29	1.000
Path Length Closed Eyes	1.27	0.0602	2.44	0.0432 *	0.54	1.000
In-line lunge	2.30	0.0433 *	2.27	0.0680	0.49	1.000

* Statistically significant *p* < 0.05.

**Table 4:** The logistic regression model regards injury occurrence.

Injury Occurrence	Variable	Odds Ratio	*p*	95% CI	Wald
General	Area Circle Closed Eyes	0.70	0.0329 *	0.50–0.97	4.55
	In-line lunge	0.49	0.0329 *	0.26–0.94	4.55
	Area Circle Closed Eyes-In-line lunge	1.40	0.0018 *	1.13–1.73	9.72
Single	Area Circle Closed Eyes	0.49	0.0094 *	0.28–0.84	6.75
	In-line lunge	0.36	0.0166 *	0.16–0.83	5.74
	Area Circle Closed Eyes-In-line lunge	1.58	0.0031 *	1.17–2.14	8.75
Multiple	Area Circle Closed Eyes	1.87	0.2830	0.60–5.84	1.15
	Path Length Closed Eyes	0.92	0.0670	0.84–1.01	3.35
	In-line lunge	0.26	0.1592	0.04–1.69	1.98
	Area Circle Closed Eyes-In-line lunge	0.80	0.4661	0.45–1.45	0.53
	Path Length Closed Eyes-In-line lunge	1.06	0.0692	1.00–1.13	3.30

* Statistically significant *p* < 0.05.

## Data Availability

The data presented in this study are available on request from the corresponding author.
